# 2-(4-Bromo­phen­yl)-3-(4-hy­droxy­phen­yl)-1,3-thia­zolidin-4-one

**DOI:** 10.1107/S1600536811025281

**Published:** 2011-07-06

**Authors:** Jasmine P. Vennila, D. John Thiruvadigal, Helen P. Kavitha, G. Chakkaravarthi, V. Manivannan

**Affiliations:** a Department of Physics, Panimalar Institute of Technology, Chennai 602 103, India; b Department of Physics, SRM University, Kattankulathur Campus, Chennai, India; c Department of Chemistry, SRM University, Ramapuram Campus, Chennai 600 089, India; d Department of Physics, CPCL Polytechnic College, Chennai 600 068, India; e Department of Research and Development, PRIST University, Vallam, Thanjavur 613 403, Tamil Nadu, India

## Abstract

In the title compound, C_15_H_12_BrNO_2_S, the dihedral angle between the two aromatic rings is 87.81 (8)°. The five-membered thia­zolidine ring has an envelope conformation, with the S atom displaced by 0.4545 (7) Å from the mean plane of the other four ring atoms. The crystal structure exhibits O—H⋯O, C—H⋯O, C—H⋯Br and C—H⋯ π inter­actions.

## Related literature

For the biolgical activity of thia­zolidine derivatives, see: Chen *et al.* (2000 ▶); Jacop & Kutty (2004 ▶); Kalia *et al.* (2007 ▶); Vicentini *et al.* (1998 ▶); Vigorita *et al.* (1992 ▶). For bond-length data see: Allen *et al.* (1987 ▶). For related structures, see: Akkurt *et al.* (2010 ▶, 2011 ▶).
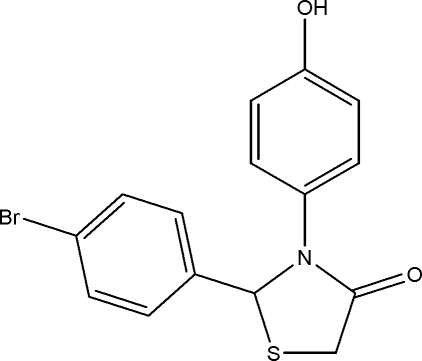


## Experimental

### 

#### Crystal data

C_15_H_12_BrNO_2_S
*M* *_r_* = 350.23
Orthorhombic, 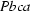

*a* = 13.3367 (6) Å
*b* = 12.6292 (5) Å
*c* = 17.1230 (6) Å
*V* = 2884.1 (2) Å^3^
*Z* = 8
Mo *K*α radiation
μ = 3.00 mm^−1^
*T* = 295 K
0.30 × 0.20 × 0.20 mm

#### Data collection

Bruker Kappa APEXII diffractometer
Absorption correction: multi-scan (*SADABS*; Sheldrick, 1996 ▶) *T*_min_ = 0.467, *T*_max_ = 0.586
18957 measured reflections
3617 independent reflections
2317 reflections with *I* > 2σ(*I*)
*R*_int_ = 0.031

#### Refinement

*R*[*F* ^2^ > 2σ(*F* ^2^)] = 0.038
*wR*(*F* ^2^) = 0.087
*S* = 1.02
3617 reflections
181 parameters
H-atom parameters constrained
Δρ_max_ = 0.45 e Å^−3^
Δρ_min_ = −0.47 e Å^−3^

### 

Data collection: *APEX2* (Bruker, 2004 ▶); cell refinement: *SAINT* (Bruker, 2004 ▶); data reduction: *SAINT*; program(s) used to solve structure: *SHELXS97* (Sheldrick, 2008 ▶); program(s) used to refine structure: *SHELXL97* (Sheldrick, 2008 ▶); molecular graphics: *PLATON* (Spek, 2009 ▶); software used to prepare material for publication: *SHELXL97*.

## Supplementary Material

Crystal structure: contains datablock(s) global, I. DOI: 10.1107/S1600536811025281/bt5562sup1.cif

Structure factors: contains datablock(s) I. DOI: 10.1107/S1600536811025281/bt5562Isup2.hkl

Supplementary material file. DOI: 10.1107/S1600536811025281/bt5562Isup3.cml

Additional supplementary materials:  crystallographic information; 3D view; checkCIF report

## Figures and Tables

**Table 1:** Hydrogen-bond geometry (Å, °) *Cg*2 is the centroid of the C4–C9 ring.

*D*—H⋯*A*	*D*—H	H⋯*A*	*D*⋯*A*	*D*—H⋯*A*
O2—H2⋯O1^i^	0.82	1.94	2.758 (2)	175
C5—H5⋯O2^ii^	0.93	2.53	3.361 (3)	149
C12—H12⋯Br1^iii^	0.93	2.84	3.463 (2)	125
C15—H15⋯*Cg*2^iv^	0.93	2.94	3.775 (2)	150

Symmetry codes: (i) 
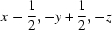
; (ii) 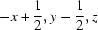; (iii) 
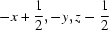
; (iv) 
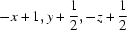
.

**Fig. 1.**
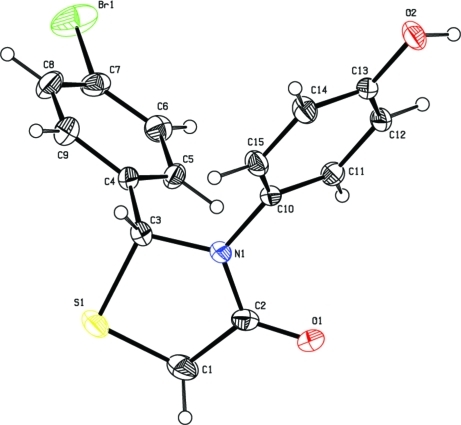
The molecular structure of (I), with atom labels and 30% probability displacement ellipsoids for non-H atoms.

**Fig. 2.**
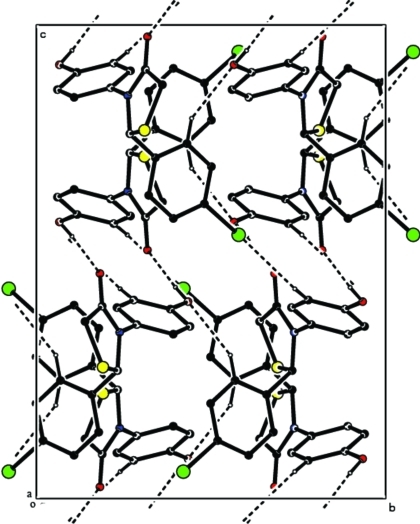
The packing of (I), viewed down the a axis. H-bonds are shown as dashed lines; H atoms not involved in hydrogen bonding have been omitted.

## supplementary crystallographic information

### Comment

Thiazolidine derivatives exhibit herbicidal (Chen *et al.*, 2000; Vicentini *et al.*, 1998), antineoplastic (Vigorita *et al.*, 1992), hypolipidemic (Jacop & Kutty, 2004) and anti-inflammatory (Kalia *et al.*, 2007) activities. We report here the crystal structure of the titled compound (I) (Fig. 1).

In the title compound (I), the bond lengths in the molecule are normal (Allen *et al.*, 1987) and in a good agreement with those reported previously (Akkurt *et al.*, 2010,2011). The dihedral angle between the benzene rings (C4—C9) and (C10—C15) is 87.81 (8)°. The five-membered thiazolidine ring (N1/C2/C1/S1/C3) has an envelope conformation, with the S atom displaced by 0.4545 (7) Å from the mean plane of the four other ring atoms. In the molecule, the absolute configuration at atom C3 is *R* configuration. The crystal structure exhibit weak O—H···O, C—H···O, C—H···Br and C—H··· π (Fig. 2 and Table 1) interactions. Cg2 is the centroid of C4-C9 ring.

### Experimental

About 5 mmol of 4-[(4-Bromobenzylidene)-amino]-phenol was taken in a round bottomed flask. To this 25 ml of dry benzene and 10 ml of mercapto acetic acid was added. The content of the flask were refluxed on the water bath for 12 hrs, cooled and poured into water. The upper organic layer was washed successively with aqueous sodium bicarbonate and water and dried with anhydrous sodium sulfate. The solvent was removed by distillation under reduced pressure. The residue on recrystallization from ethanol several times, yielded diffraction quality crystals.Yield: 62%.

### Refinement

The C-bound H atoms were geometrically placed (C—H = 0.93–0.98 Å) and refined as riding with *U*_iso_(H) = 1.2Ueq(C) and O—H = 0.82 Å and *U*_iso_(H) = 1.2Ueq(O) for OH.

### Crystal data

**Table:**

C_15_H_12_BrNO_2_S	*F*(000) = 1408
*M**_r_* = 350.23	*D*_x_ = 1.613 Mg m^−^^3^
Orthorhombic, *P**b**c**a*	Mo *K*α radiation, λ = 0.71073 Å
Hall symbol: -P 2ac 2ab	Cell parameters from 4654 reflections
*a* = 13.3367 (6) Å	θ = 2.4–23.8°
*b* = 12.6292 (5) Å	µ = 3.00 mm^−^^1^
*c* = 17.1230 (6) Å	*T* = 295 K
*V* = 2884.1 (2) Å^3^	Block, colourless
*Z* = 8	0.30 × 0.20 × 0.20 mm

### Data collection

**Table:**

Bruker Kappa APEXII diffractometer	3617 independent reflections
Radiation source: fine-focus sealed tube	2317 reflections with *I* > 2σ(*I*)
graphite	*R*_int_ = 0.031
ω and φ scans	θ_max_ = 28.4°, θ_min_ = 2.4°
Absorption correction: multi-scan (*SADABS*; Sheldrick, 1996)	*h* = −17→16
*T*_min_ = 0.467, *T*_max_ = 0.586	*k* = −16→16
18957 measured reflections	*l* = −16→22

### Refinement

**Table:**

Refinement on *F*^2^	Primary atom site location: structure-invariant direct methods
Least-squares matrix: full	Secondary atom site location: difference Fourier map
*R*[*F*^2^ > 2σ(*F*^2^)] = 0.038	Hydrogen site location: inferred from neighbouring sites
*wR*(*F*^2^) = 0.087	H-atom parameters constrained
*S* = 1.02	*w* = 1/[σ^2^(*F*_o_^2^) + (0.0332*P*)^2^ + 1.5996*P*] where *P* = (*F*_o_^2^ + 2*F*_c_^2^)/3
3617 reflections	(Δ/σ)_max_ < 0.001
181 parameters	Δρ_max_ = 0.45 e Å^−^^3^
0 restraints	Δρ_min_ = −0.47 e Å^−^^3^

### Fractional atomic coordinates and isotropic or equivalent isotropic displacement parameters (Å^2^)

**Table:**

	*x*	*y*	*z*	*U*_iso_*/*U*_eq_	
C1	0.63473 (19)	0.1392 (2)	0.12609 (17)	0.0510 (7)	
H1A	0.6916	0.1565	0.0932	0.061*	
H1B	0.6280	0.0628	0.1279	0.061*	
C2	0.54086 (16)	0.18813 (18)	0.09361 (15)	0.0344 (5)	
C3	0.52231 (15)	0.23237 (19)	0.22967 (13)	0.0328 (5)	
H3	0.5202	0.3035	0.2525	0.039*	
C4	0.46182 (16)	0.15917 (18)	0.28031 (13)	0.0316 (5)	
C5	0.41708 (17)	0.06902 (18)	0.25054 (14)	0.0342 (5)	
H5	0.4201	0.0555	0.1972	0.041*	
C6	0.36796 (18)	−0.00109 (19)	0.29911 (15)	0.0387 (6)	
H6	0.3372	−0.0612	0.2788	0.046*	
C7	0.36517 (19)	0.0191 (2)	0.37752 (15)	0.0427 (6)	
C8	0.4075 (2)	0.1078 (2)	0.40845 (16)	0.0515 (7)	
H8	0.4044	0.1204	0.4619	0.062*	
C9	0.4548 (2)	0.1784 (2)	0.35973 (15)	0.0459 (7)	
H9	0.4825	0.2399	0.3804	0.055*	
C10	0.39180 (15)	0.28732 (16)	0.13260 (12)	0.0262 (5)	
C11	0.31686 (16)	0.23068 (17)	0.09663 (13)	0.0305 (5)	
H11	0.3274	0.1603	0.0830	0.037*	
C12	0.22563 (16)	0.27854 (18)	0.08068 (13)	0.0324 (5)	
H12	0.1745	0.2400	0.0571	0.039*	
C13	0.21067 (15)	0.38358 (18)	0.09987 (13)	0.0313 (5)	
C14	0.28555 (17)	0.43972 (18)	0.13648 (15)	0.0373 (6)	
H14	0.2751	0.5101	0.1502	0.045*	
C15	0.37581 (17)	0.39194 (18)	0.15282 (14)	0.0351 (5)	
H15	0.4263	0.4301	0.1776	0.042*	
N1	0.48688 (12)	0.23846 (14)	0.14893 (10)	0.0290 (4)	
O1	0.51648 (12)	0.18202 (13)	0.02472 (10)	0.0432 (4)	
O2	0.12206 (12)	0.43423 (13)	0.08407 (11)	0.0472 (5)	
H2	0.0887	0.3982	0.0539	0.057*	
S1	0.65353 (5)	0.19057 (6)	0.22246 (4)	0.04894 (19)	
Br1	0.29816 (3)	−0.07918 (3)	0.44309 (2)	0.07795 (16)	

### Atomic displacement parameters (Å^2^)

**Table:**

	*U*^11^	*U*^22^	*U*^33^	*U*^12^	*U*^13^	*U*^23^
C1	0.0342 (14)	0.0612 (17)	0.0575 (18)	0.0169 (13)	0.0023 (12)	0.0021 (14)
C2	0.0277 (12)	0.0379 (12)	0.0376 (16)	0.0002 (10)	0.0076 (11)	0.0026 (11)
C3	0.0271 (11)	0.0393 (12)	0.0320 (14)	0.0020 (10)	−0.0051 (10)	−0.0013 (10)
C4	0.0285 (11)	0.0418 (13)	0.0244 (13)	0.0066 (10)	−0.0036 (9)	−0.0008 (10)
C5	0.0393 (13)	0.0402 (12)	0.0229 (12)	0.0040 (11)	−0.0011 (10)	0.0000 (11)
C6	0.0402 (14)	0.0346 (13)	0.0413 (16)	0.0070 (11)	0.0006 (11)	0.0037 (11)
C7	0.0436 (14)	0.0497 (15)	0.0349 (16)	0.0170 (12)	0.0104 (12)	0.0136 (13)
C8	0.0581 (18)	0.0711 (19)	0.0254 (14)	0.0138 (15)	0.0042 (13)	−0.0003 (14)
C9	0.0483 (15)	0.0560 (16)	0.0335 (16)	0.0013 (13)	−0.0020 (12)	−0.0098 (13)
C10	0.0226 (10)	0.0330 (11)	0.0230 (12)	0.0013 (9)	0.0013 (9)	0.0011 (9)
C11	0.0320 (12)	0.0290 (11)	0.0305 (13)	0.0008 (10)	0.0002 (10)	−0.0040 (10)
C12	0.0258 (11)	0.0405 (13)	0.0310 (13)	−0.0017 (10)	−0.0041 (9)	−0.0098 (10)
C13	0.0253 (11)	0.0421 (12)	0.0265 (13)	0.0064 (10)	−0.0012 (9)	−0.0062 (10)
C14	0.0344 (13)	0.0331 (12)	0.0442 (15)	0.0038 (10)	−0.0071 (11)	−0.0118 (11)
C15	0.0264 (11)	0.0388 (13)	0.0400 (15)	−0.0039 (10)	−0.0059 (10)	−0.0062 (11)
N1	0.0234 (9)	0.0362 (10)	0.0273 (11)	0.0037 (8)	−0.0009 (8)	0.0024 (8)
O1	0.0371 (9)	0.0622 (11)	0.0304 (11)	0.0067 (8)	0.0078 (8)	−0.0037 (8)
O2	0.0324 (9)	0.0557 (11)	0.0534 (11)	0.0145 (8)	−0.0157 (8)	−0.0223 (9)
S1	0.0268 (3)	0.0699 (5)	0.0501 (5)	0.0060 (3)	−0.0060 (3)	0.0099 (4)
Br1	0.1055 (3)	0.0617 (2)	0.0667 (3)	0.02192 (19)	0.0415 (2)	0.03136 (17)

### Geometric parameters (Å, °)

**Table:**

C1—C2	1.503 (3)	C7—Br1	1.897 (2)
C1—S1	1.791 (3)	C8—C9	1.375 (4)
C1—H1A	0.9700	C8—H8	0.9300
C1—H1B	0.9700	C9—H9	0.9300
C2—O1	1.226 (3)	C10—C11	1.375 (3)
C2—N1	1.349 (3)	C10—C15	1.382 (3)
C3—N1	1.463 (3)	C10—N1	1.438 (3)
C3—C4	1.502 (3)	C11—C12	1.386 (3)
C3—S1	1.832 (2)	C11—H11	0.9300
C3—H3	0.9800	C12—C13	1.381 (3)
C4—C5	1.383 (3)	C12—H12	0.9300
C4—C9	1.385 (3)	C13—O2	1.371 (3)
C5—C6	1.380 (3)	C13—C14	1.376 (3)
C5—H5	0.9300	C14—C15	1.375 (3)
C6—C7	1.367 (3)	C14—H14	0.9300
C6—H6	0.9300	C15—H15	0.9300
C7—C8	1.361 (4)	O2—H2	0.8200
			
C2—C1—S1	107.98 (18)	C7—C8—H8	120.4
C2—C1—H1A	110.1	C9—C8—H8	120.4
S1—C1—H1A	110.1	C8—C9—C4	120.9 (2)
C2—C1—H1B	110.1	C8—C9—H9	119.6
S1—C1—H1B	110.1	C4—C9—H9	119.6
H1A—C1—H1B	108.4	C11—C10—C15	119.83 (19)
O1—C2—N1	124.3 (2)	C11—C10—N1	120.33 (19)
O1—C2—C1	123.5 (2)	C15—C10—N1	119.84 (19)
N1—C2—C1	112.2 (2)	C10—C11—C12	120.0 (2)
N1—C3—C4	113.86 (18)	C10—C11—H11	120.0
N1—C3—S1	105.06 (14)	C12—C11—H11	120.0
C4—C3—S1	111.99 (15)	C13—C12—C11	119.9 (2)
N1—C3—H3	108.6	C13—C12—H12	120.0
C4—C3—H3	108.6	C11—C12—H12	120.0
S1—C3—H3	108.6	O2—C13—C14	118.4 (2)
C5—C4—C9	118.5 (2)	O2—C13—C12	121.7 (2)
C5—C4—C3	121.7 (2)	C14—C13—C12	119.9 (2)
C9—C4—C3	119.7 (2)	C15—C14—C13	120.1 (2)
C6—C5—C4	120.7 (2)	C15—C14—H14	119.9
C6—C5—H5	119.6	C13—C14—H14	119.9
C4—C5—H5	119.6	C14—C15—C10	120.2 (2)
C7—C6—C5	119.0 (2)	C14—C15—H15	119.9
C7—C6—H6	120.5	C10—C15—H15	119.9
C5—C6—H6	120.5	C2—N1—C10	122.44 (18)
C8—C7—C6	121.6 (2)	C2—N1—C3	117.80 (18)
C8—C7—Br1	120.2 (2)	C10—N1—C3	119.42 (17)
C6—C7—Br1	118.2 (2)	C13—O2—H2	109.5
C7—C8—C9	119.2 (2)	C1—S1—C3	91.88 (11)
			
S1—C1—C2—O1	168.02 (19)	O2—C13—C14—C15	−179.5 (2)
S1—C1—C2—N1	−12.4 (3)	C12—C13—C14—C15	1.1 (4)
N1—C3—C4—C5	32.4 (3)	C13—C14—C15—C10	0.0 (4)
S1—C3—C4—C5	−86.6 (2)	C11—C10—C15—C14	−0.6 (3)
N1—C3—C4—C9	−151.3 (2)	N1—C10—C15—C14	179.1 (2)
S1—C3—C4—C9	89.7 (2)	O1—C2—N1—C10	2.4 (3)
C9—C4—C5—C6	−1.1 (3)	C1—C2—N1—C10	−177.1 (2)
C3—C4—C5—C6	175.3 (2)	O1—C2—N1—C3	175.7 (2)
C4—C5—C6—C7	−0.8 (3)	C1—C2—N1—C3	−3.9 (3)
C5—C6—C7—C8	1.6 (4)	C11—C10—N1—C2	52.6 (3)
C5—C6—C7—Br1	−179.47 (17)	C15—C10—N1—C2	−127.1 (2)
C6—C7—C8—C9	−0.5 (4)	C11—C10—N1—C3	−120.6 (2)
Br1—C7—C8—C9	−179.4 (2)	C15—C10—N1—C3	59.7 (3)
C7—C8—C9—C4	−1.5 (4)	C4—C3—N1—C2	−105.3 (2)
C5—C4—C9—C8	2.2 (4)	S1—C3—N1—C2	17.6 (2)
C3—C4—C9—C8	−174.2 (2)	C4—C3—N1—C10	68.2 (3)
C15—C10—C11—C12	0.1 (3)	S1—C3—N1—C10	−168.91 (15)
N1—C10—C11—C12	−179.6 (2)	C2—C1—S1—C3	18.91 (19)
C10—C11—C12—C13	1.0 (3)	N1—C3—S1—C1	−20.26 (17)
C11—C12—C13—O2	179.1 (2)	C4—C3—S1—C1	103.83 (18)
C11—C12—C13—C14	−1.6 (4)		

### Hydrogen-bond geometry (Å, °)

**Table:**

Cg2 is the centroid of the C4–C9 ring.

**Table:**

*D*—H···*A*	*D*—H	H···*A*	*D*···*A*	*D*—H···*A*
O2—H2···O1^i^	0.82	1.94	2.758 (2)	175
C5—H5···O2^ii^	0.93	2.53	3.361 (3)	149
C12—H12···Br1^iii^	0.93	2.84	3.463 (2)	125
C15—H15···Cg2^iv^	0.93	2.94	3.775 (2)	150

Symmetry codes: (i) *x*−1/2, −*y*+1/2, −*z*; (ii) −*x*+1/2, *y*−1/2, *z*; (iii) −*x*+1/2, −*y*, *z*−1/2; (iv) −*x*+1, *y*+1/2, −*z*+1/2.
